# Wanna See My Dog Pic? A Comparative Observational Study of the Presentation of Animals on Online Dating Profiles in Vienna and Tokyo

**DOI:** 10.3390/ani12030230

**Published:** 2022-01-19

**Authors:** Christian Dürnberger, Svenja Springer

**Affiliations:** Messerli Research Institute, Ethics and Human–Animal Studies, University of Veterinary Medicine Vienna, Veterinaerplatz 1, 1210 Vienna, Austria; Svenja.springer@vetmeduni.ac.at

**Keywords:** online dating application, dogs, cats, human–animal relationship, observational study

## Abstract

**Simple Summary:**

Online dating applications offer new ways for people to search for social contacts. While previous studies have indicated that the inclusion of animals in profiles can increase users’ dating success rates, the question of how many users display animals, and what kinds of animal are shown on dating profiles, has not yet been empirically investigated. Using a structured observational study of profiles in Vienna and Tokyo on a popular online dating app (*n* = 2400), we examined how many profiles show animals and what kinds of animal are shown. Further, we investigated whether cultural background (Vienna versus Tokyo), gender, age and sexual orientation affect the use of animal pictures. Approximately 16% of investigated profiles had at least one photo showing an animal. In both cities, dogs were the most frequently shown animal, followed by cats. Further, results indicate that users in Vienna, women and older users were more likely to present animals on their profiles.

**Abstract:**

Online dating applications offer new ways for people to search for social contacts. While previous studies have indicated that the inclusion of animals in profiles can increase users’ dating success rates, the question of how many users display animals, and what kinds of animals are shown on dating profiles, has not yet been empirically investigated. Using a structured observational study of profiles in Vienna and Tokyo on a popular online dating app (*n* = 2400), we therefore looked at how many profiles show animals and what kinds of animals are shown. We found that 15.5% of the investigated profiles had at least one photo showing an animal. In both cities, dogs were the most frequently shown animal. Taking the cities together, they appeared in 46.4% of the animal pictures, as compared with cats at 25.7%. Other animals such as exotic animals (9.9%), farm animals (6.4%) or horses (4.6%) played a minor role. Users were significantly more likely to show cats in Tokyo (35.8%) than they were in Vienna (18.0%). We found that users in Vienna; women; and older adults were more likely to present animals on their profiles than were users in Tokyo; men; and younger users. Sexual orientation showed no significant differences in the analyses.

## 1. Introduction

In recent years, the increasing use of online dating platforms and applications (from here on “apps”) has led to at least two decisive developments: first, a change in the way in which people search for social contacts, casual encounters or committed relationships; and second, a new opportunity for users of the apps to present themselves in ways that make a positive impression. Against this background, the present study combines two themes of growing significance in contemporary society: online dating apps, and the role and integration of animals in human life.

In 2015, 185 million people used dating apps; in 2020, the number of global users rose to 270 million, and the number is continuously increasing [1]. In this process, various dating apps have been established, all of which have the main goal of connecting people in a simple, convenient way. With an average of 66 million active users a month [2], Tinder^®^ is currently considered the most popular app, and it dominates the dating market worldwide [1,2]. Founded in 2012, this app has revolutionised the dating industry with its simple-to-use design. By using smartphone GPS functionalities, it searches for people who are located in the same region and suggests potentially suitable dating candidates based on gender, sexual orientation and age. In addition, users are able to upload several profile photos or write short profile texts about themselves. With this information, the user can make a decision: swiping right means “like”, swiping left means “pass”. When two users “like” each other, this produces a “match”, meaning that they are able to chat with each other from then on via the app. Although one can add a short, written self-description, the mechanism and design of the app emphasises visual impression—it encourages users to focus on profile photos [3,4]. This visual focus is reflected in the way people actually make use of the app. For instance, previous studies revealed that profile texts are rarely, if ever, considered by users, who mainly, or exclusively, make their decisions on the basis of the visual impression given by the (first) profile photo(s) [5,6].

Although online dating apps are sometimes called “hook-up” or “sex” apps [1], the motives of those who use them seem to be more diverse. As research indicates, these apps are used not only as a searching tool for casual sex but also by those looking for relationships, entertainment, thrills and excitement, new social contacts, an ego-boost or just to be trendy [5,7,8,9]. However, the aims to control the way one is represented to others [10,11] and convey a positive impression unite all users [5]. Although it can be argued that users of online dating profiles tend toward overstatement, research indicates that users look for a balance between an ideal and authentic self-presentation [5,10]. Degen and Kleeberg-Niepage [4] and Ward [5] have emphasised that users’ self-presentation is not a spontaneous act but a rather conscious choice they make. So, users of online dating apps think about how they are going to present themselves, and in particular about what photos to upload, to make a lasting and positive impression on other users. As Jagger noted more generally: “Dating advertisements are a revealing site for examining the social construction of identities—identities that are deemed desirable and marketable in a specific cultural context” [12] (p. 90).

With this in mind, users are encouraged to make the right visual impressions in order to present themselves favourably. For instance, users with an interest in sports or traveling will probably make these visible by showing themselves doing sports or traveling in foreign countries. Besides hobbies, a further interest or passion can be easily shown via profile photos: namely, the user’s interest in, and passion for, animals. Undoubtedly, in recent decades, especially, the role and perception of animals has changed, both in society and science. In particular, our handling of companion animals, and the integration of them in our lives, signal the changing human–animal relationship [13]. Animals such as dogs and cats are increasingly viewed and treated as family members, and tight emotional relationships between animals and their owners, or people in general, can be observed [13,14]. Relatedly, the term animal turn indicates the increased scholarly interest in animals in various academic disciplines. For instance, the field of human–animal studies investigates multiple and complex relationship forms between humans and animals in an interdisciplinary way. It includes disciplines such as history, philosophy, literary studies, communications sciences and sociology [15].

Against this background, it comes as no surprise that our spatial proximity and emotional closeness to companion animals is also evident in the special situation of dating. Guéguen and Ciccotti [16] have shown that men are more successful in obtaining women’s phone numbers in encounters on the street when they are accompanied by a dog than they are without one. Since dogs are usually perceived as social animals that need the attention and care of their owner, they seem to reflect owner characteristics such as being social, reliable or empathic, which in turn have a positive effect on dating [17]. In addition, research indicates differences between animal species [18,19,20]. For instance, heterosexual men labelled as “dog persons” may be perceived as more masculine and attractive than they are when labelled “cat persons” [19,20]. This does not necessarily mean that photos with animals always improve the chances of success. For example, Kogan and Volsche [18] surveyed 708 women aged 18–24 concerning the impact of cats on the dateability of men. They found that men with cats were perceived as less masculine by female respondents, representing a higher level of neuroticism, and hence were perceived as less dateable for both short- and long-term pairing [18]. 

Whereas previous publications have focused on why animals appear on dating profiles, and the extent animals to which they increase the chances of success [16,17,19,20], the question of how many users of online dating platforms display animals, and of what kind of animals are shown, has not, as far as we know, been empirically investigated. Given this, the overall aim of the present observational study of profiles on a popular online dating app in Vienna and Tokyo was to examine the prevalence of users showing animals on their dating profile and to investigate whether differences in socio-demographic factors, including different cultural backgrounds, can be overserved.

A German study showed that around one quarter (26%) of animal owners are 60 years or older, whereas only 15% are younger than 30 years [21]. In addition, differences between men and women were found. There was evidence that women are more likely to keep animals than men [22]. Assuming that people who own animals are more likely to present them on their profiles than those that do not, it is to be expected that more women, and older users, will picture animals such as dogs and cats on their profiles than men and younger users. Whereas these German studies [21,22] build an appropriate basis for the development of hypotheses for the Austrian context, these numbers might differ in Japan. However, to the authors’ best knowledge, studies on this issue are not available for the Japanese context. Further, it is noticeable that most existing research into the use of dating apps and related issues has focused on heterosexual relationships [16,17,19,20]. The present study attempts to overcome this limitation by considering both male and female heterosexual and homosexual users.

To determine whether cultural differences in the presentation of animals on the selected app exist, profiles were analysed from Vienna, exemplifying a metropolitan area in Central Europe, and Tokyo, a metropolitan area in Asia. These cities were chosen because they may be considered culturally distinct in their social norms [23] and because their citizens have rather different possibilities of integrating animals in their lives. In 2020, for instance, 55,649 dogs were registered in Vienna, and their numbers are rising [24]. The provision of dog zones in every district, and individual sections in public parks designated for dog owners with their animals, makes Vienna especially attractive for dog owners [24]. By contrast, in Tokyo dog numbers are falling somewhat [25]. Strict regulation in public areas as well as the quite common prohibition requiring owners to keep their animals in their apartments make it less attractive to keep animals in this city. It is likely that these different circumstances would lead to users in Vienna showing a dog or a cat on their profile photos more often than users in Tokyo would. On the other hand, a major trend in Tokyo is the establishment of cat cafes. It is estimated that there are 40 such cafes in the city. Although the number of animals might be lower in Tokyo, it is likely that cats, especially, as opposed to dogs, will be shown in online dating profile photos there. Based on data from an observational study of profiles from Vienna and Tokyo on the selected online dating app, it is the overall purpose of this paper to empirically address the following research questions. (i) How many profiles show animal photos, and do socio-demographic factors explain differences between profiles with and without animals? (ii) Where animals are pictured, what animal species are shown, and are there differences here between users from Vienna and Tokyo? Finally, (iii) to what extent do socio-demographic factors and the total number of profile photos have an impact on the likelihood that users are (a) including pictures of animals in general, (b) using a photo with/of an animal as their first profile picture and (c) whether a cat or dog is displayed on one of the profile photos?

## 2. Material and Methods

### 2.1. Cross-Sectional Structured Observational Study and the Role of the Observer

The cross-sectional structured observational study [26] aimed to gather quantitative data on the presentation of animal photos on Tinder^®^ (Match Group, Inc., Dallas, TX, USA) profiles. The researcher (C.D.) had the status of an independent scientific observer who collected data on socio-demographic factors of app-users and the presentation of animal photos on user profiles. This was an observation with a low degree of participation since the researcher took a passive role without making any contact with the other users at any point in the study. Data were collected based on information from profiles only. At no point did the researcher “like” profiles or answer received “likes” from other users. In addition, the observation was covert, as the observed users were unaware that the researcher profile was created by a research group.

The study was approved by the head of the Ethics Committee of the Medical University of Vienna (Ref: 1698_001) and the chief privacy officer of the Vetmeduni, Vienna. 

### 2.2. Research Account and Set-Up of Online Dating Profiles

A “Tinder^®^ plus” account was set up for data collection. This type of account allows users to search globally for other users regardless of where they are located. In total, eight chronologically consecutive profiles were set up for data collection: four for Vienna and four for Tokyo. Table 1 shows characteristics of the profiles designed for this study, including city, name of designed profile, gender, sexual orientation and age (see Table 1). Since the selected app captures gender and sexual orientation in binaries, both variables were gathered as dichotomous binaries. 

Since the selected dating app put an emphasis on profile photos, the same photo was uploaded for each research profile displaying Saint Stephen’s Cathedral in Vienna. This rather neutral photo was chosen to avoid giving false impression and false expectations to other users who came across the research profile while using the dating app.

### 2.3. Selection Process and Search Criteria for Observation

The target and source population for this observational study were users of the selected online dating app, and the users eligible for inclusion in the study needed to have at least one uploaded profile photo [27]. In total, 2400 Tinder^®^ profiles were examined (300 profiles per research profile). Based on the set-up options provided by the app, gender (female/male), sexual orientation (heterosexual/homosexual), age (in years) and distance (in km) were the relevant criteria for the stratified sampling. Regardless of whether or not these profiles showed an animal, socio-demographic data were collected, and the numbers of profile photos were noted. However, the main target group of participants was those users who displayed animals and hence would contribute data on the investigated research questions. The data were collected in the period 16–22 March 2021. The eight research profiles set up reflected differences in gender, sexual orientation, age and search distance in km (see Table 2).

### 2.4. Development of the Structured Observational Study

A set of questions was developed based on a preliminary investigation of the profiles with the aim of gaining quantitative data on the prevalence of animal photos on the selected online dating app. This preliminary investigation was of relevance to identify what information is provided on the app, since this has an impact on what questions, in relation to socio-demographic aspects or photos, can be answered in the course of the study. As a consequence, questions and codes were created related to the information provided by profiles. The sets of questions and codes were piloted against ten profiles showing animals. This pilot served to check (i) that the sequence of questions was practicable; (ii) that the animals were categorised in the same way by both researchers (C.D., S.S.), ensuring inter-rater reliability; and (iii) that the structure and content of questions were suitable for the observational study. Based on results from the pilot, minor adjustments and changes were made in order to improve the practicability of the questionnaire and the content of the questions. In case of uncertainty in relation to the categorisation of animals, the responsible researcher for data collection (C.D.) was in exchange with the second researcher involved in this project (S.S.) in order to ensure consistent and appropriate categorisation of animals during the entire period of data collection. 

### 2.5. Study Design and Measurements

The questionnaire for study consisted of three sections. Section A included three closed-ended questions about sexual orientation, age and number of uploaded profile photos. Section B requested information on the animals presented in the profile photos (see Table 3).

To ensure the investigated profiles in Vienna and Tokyo were comparable, the categorisation into farm animals, wild animals and exotic animals was *always* based on the Austrian context. Thus, for instance, regardless of whether an eagle appeared on an Austrian or Japanese profile, in Vienna or in Tokyo, the animal was categorised as a wild animal since eagles are native to Austria’s wilderness. Whales, in contrast, were always categorised—again, based on the Austrian context—as an exotic animal, even though they are considered wild animals in Japan. The third section, C, focused on the written profile texts of the users. The data from section C are not relevant to the research questions considered in this paper and are therefore not presented here. 

In addition, a research diary [28] was used in which the observer (C.D.) recorded notes during data collection. The aim of the diary was to collect impressions of investigated profile photos that would not be revealed by the set of closed-ended questions. Hence, these notes provided a qualitative view of the data going beyond the quantitative findings, which may serve as a basis for the development of hypotheses for future research studies.

### 2.6. Data Collection and Analyses

The questionnaire was designed using the survey software Alchemer^®^ (Alchemer^®^, Louisville, CO, USA). Univariate descriptive statistics were presented in tables, figures or text. For bivariate analysis, χ^2^-tests were conducted to assess whether the frequency distribution of gender and city, “Animal(s) on profile photo(s)”, “Animal(s) on the first profile photo” and “Classified animal species” differed in the sub-populations in Vienna and Tokyo. Mann–Whitney-U Tests were conducted with the variable age, number of profile photos (continuous variables) and age groups (ordinal scaled variable) to detect differences between (a) Vienna and Tokyo and (b) profiles with animals and without animals. Spearman’s rho correlations were calculated to identify whether associations between the (i) number of profile photos, (ii) age of user and (iii) number of photos displaying animals were associated.

We then pooled the data from both cities and ran three binary logistic regression models to understand how changes in values of the socio-demographic factors were associated with changes in the probability of the following outcomes: (a) users are displaying animals in general on photos, (b) users are displaying animals on their first profile photo, and (c) a cat or dog is displayed on a photo. The dependent variables were inserted on a dichotomous scale. The scale for (a) and (b) was: 1 = yes; 2 = no, and the scale for (c) was: 1 = dog; 2 = cat. Categorical predictor variables inserted in the regression analyses were gender (1 = male, 2 = female), city (1 = Vienna, 2 = Tokyo) and sexual orientation (1 = heterosexual; 2 = homosexual). Age and number of profile photos were included as continuous predictor variables. IBM^®^ SPSS^®^ Statistics version 26.0 (IBM^®^ SPSS^®^ Statistics, Chicago, IL, USA) was used in all analyses. The significance level was 0.05.

## 3. Results

### 3.1. Overview of Socio-Demographic Results

In total, 2400 profiles of the selected online dating app were included in the study. Using the research profiles, we created (see Table 1) we collected data from 300 heterosexual and homosexual men, and 300 heterosexual and homosexual women, in Vienna and Tokyo. With a mean age of 28.6 ± 4.5 years, users in Vienna were significantly older than users in Tokyo (27.0 ± 5.1; *p* < 0.001). Further, the users of the investigated profiles in Vienna showed significantly more photos on their profiles than those in Tokyo (*p* < 0.001). In Tokyo, a positive correlation was identified between user age and number of profile photos (r_s_ = 0.134, *p* < 0.001) (see Table 4).

### 3.2. Comparison between Profiles with Animals and Profiles without Animals

Of the 2400 investigated profiles, 373 (15.5%) displayed at least one animal photo. In both cities, we found a positive correlation between the number of profile photos and the number of profile photos showing animals (Vienna: r_s_ = 0.184; *p* = 0.008 | Tokyo: r_s_ = 0.206; *p* = 0.009). 

Comparison of the users who displayed animal photos on their profile and the users who did not do so resulted in the following significant differences (see Table 5). On the selected analysed dating app, significantly more women than men (*p* = 0.049) present animal photos on their profiles. Further, significantly more users in Vienna (*p* = 0.006), and significantly more older users (*p* = 0.019), have profiles with animal photos as compared with users in Tokyo and younger users. In addition, users who display an animal photo on their profile post, on average, display one more photo than users who do not do so (*p* < 0.001). No significant differences between heterosexual and homosexual users of the analysed app were identified (*p* = 0.639) (see Table 5).

### 3.3. Prevalence and Categorisation of Animals Displayed on Profiles

A further aim of the study was to determine how many profiles showed animals and what kinds of animal were presented. In general, significantly more users in Vienna (211; 17.6%) show animals on their profile than users in Tokyo (162; 13.5%) (χ^2^(1) = 7.622; *p* = 0.006). Most of the profiles—i.e., 77.7% in Vienna and 76.5% in Tokyo—showed the animal, or animals, on just one profile photo. In a smaller proportion of cases—i.e., 22.3% in Vienna and 23.5% in Tokyo—the users had more than one photo showing the animal, or animals, in their profile.

#### 3.3.1. Presentation of Animals in the First Profile Photo

Of the 373 users choosing to include animal photos, 73 (19.6%) displayed the animals on their first profile photo. Here, comparison of users in Vienna and Tokyo revealed significant differences as 65.9% users in Vienna showed a dog on the first photo as compared with 31.3% of users in Tokyo (χ^2^(1) = 8.610, *p* = 0.003). In addition to that, only profiles in Vienna (12.2%) have shown farm animals on the first profile photo. This resulted in a significant difference to users in Tokyo (χ^2^(1) = 4.189, *p* = 0.041). We also found that more users in Tokyo displayed cats (40.6%) and exotic animals (15.6%) in their first profile photos than users in Vienna (cats = 2.4%; exotic animals = 0.0%) (cats: χ^2^(1) = 7.819, *p* = 0.005; exotic animals: χ^2^(1) = 6.877, *p* = 0.009).

#### 3.3.2. Presentation of Animals in All Profile Photos (Including the First Profile Photo)

**Figure 1:**
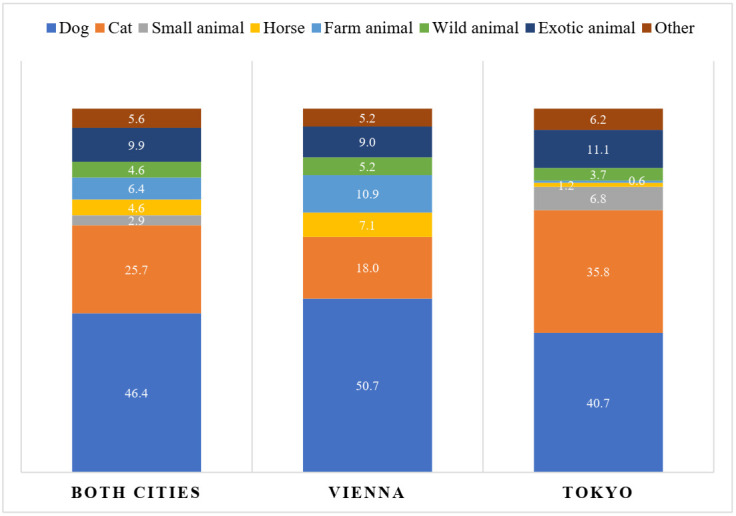
Percentages of animal types shown on all profile photos (including the first profile photo) for both cities (*n* = 373), Vienna (*n* = 211) and Tokyo (*n* = 162); category “other” includes cagebirds, aquarium, terrarium, meme and others.

Figure 1 shows the percentages of various animal species shown on the analysed profiles. Again, comparison between the profiles in Vienna and Tokyo revealed significant differences here. Users in Tokyo were significantly more likely to show cats (35.8%) and small animals (6.8%) than users in Vienna (cats = 18.0%; small animals = 0.0%) (cats: χ^2^(1) = 15.179, *p* < 0.001; small animals: χ^2^(1) = 14.763, *p* < 0.001). The Viennese profiles included farm animal (10.9%) and horse (7.1%) photos significantly more often than the profiles in Tokyo (farm animals = 0.6%; horses = 1.2%) (farm animals: χ^2^(1) = 16.096, *p* < 0.001; horses: χ^2^(1) = 7.270, *p* = 0.007) (see Figure 1).

### 3.4. Effects of Socio-Demographic Factors and Number of Profile Photos on Posting Animal Photos

We pooled data from Vienna and Tokyo and conducted three binary logistic regression models in order to determine whether socio-demographic factors or total numbers of profile photos were associated with the decision of the user (i) to show an animal, or animals, at all; (ii) to show an animal, or animals, on the first photo; or (iii) to show a dog rather than a cat (or vice versa) (see Table S1). 

We found that users of the analysed app with several profile photos, and women, are more likely to display an animal on their profile photo than users with fewer photos (χ^2^(1) = 86.798; *p* < 0.001) and men (χ^2^(1) = 3.942; *p* = 0.047). Additionally, being a younger user (χ^2^ (1) = 9.315, *p* = 0.002) and having a higher number of profile photos (χ^2^(1) = 17.462; *p* < 0.001) increased the probability of the animal being shown in the first profile photo. Significantly more often than users in Tokyo, Viennese users displayed photos of dogs, but where cats were concerned the reverse was found to be the case (χ^2^(1) = 14.281; *p* < 0.001).

### 3.5. Qualitative Notes in Research Diary

Notes were taken with the aim of gaining qualitative insights into the data, and specifically developed preliminary conclusions about how animals are presented on the investigated profiles. The notes suggested that two typical types of presentation of animals (including sub-categories) could be identified. These types were not mutually exclusive. However, they provide an initial overview of the ways in which animals are presented on the analysed online dating app, and they offer a basis on which to develop category systems for future investigations.

(i) The animal as close friend and/or family member: this form of presentation includes photos showing a portrait of an animal. The animal is prominently displayed, and the impression given is that the animal is as “deserving” of its own photo in the gallery of the user as are children or close friends. Its inclusion is part of describing one’s life and important companions in photos.
(ii) The animal as a mirror of the user’s personal character traits: this type of photo is not about the animal per se. Instead, the form of presentation gives certain insights into the life and character traits of the user. In total, four sub-categories of this form of presentation were identified:
  (a) The animal as a part of an emotional encounter: A typical photo shows a user cuddling with a dog or cat in bed.
  (b) The animal as part of an active and/or sporty lifestyle: here, animals are used to indicate activity and a healthy, sporty and active lifestyle through their presentation in the context of outdoor activities. A typical photo shows a user jogging with a dog or looking at a cow while hiking.
  (c) The animal as part of aesthetics: in this context, animals are used to communicate the user’s fashion or housing style. A typical photo shows a white dog with a turquoise hair band on the couch in a flat where all the design elements are white or turquoise.
  (d) The animal as part of humour: here, animals appear humorously staged. A typical photo shows a cat with an amusing hairstyle or with its eyes wide open as if amazed at what it sees. Photos in this category are intended not so much to show the user’s own animals but rather as internet memes.

## 4. Discussion

The present study provides the first systematically collected data on the prevalence of animal photos on profiles on a popular online dating app. The data indicated that around 16% of the investigated profiles displayed at least one animal. Due to a lack of data on this issue, it is difficult to assess this number against the background of previous studies. However, in recent years, a lively debate about the presentation of animals on online dating apps has taken place in media such as newspapers and online blogs [29,30,31,32]. In these media, reports and articles discuss the role of animals in online dating and ask specifically whether animals improve dating success rates, as Guéguen and Ciccotti [16] note. Based on the high level of media interest in this topic, the percentage of animals in profile photos might have been expected to be higher than 16%. Relatedly, Watson [31] uses the term “dogfishing” to mark the fact that it is not only dog owners who pose on their online dating profile with animals but also users who do not own a dog but believe that showing one will attract more matches. However, given the relatively low prevalence of animal photos on the analysed profiles we found, it seems questionable whether this occurs often in reality or is rather something hyped up by the media. If the phenomenon were frequent, we would surely expect the number of photos displaying animals to be higher.

Turning to the question of what kinds of animal appear on the investigated online dating profiles, our evidence confirms that it is mainly dogs and cats that are shown. Here, differences between Tokyo and Vienna were identified. At approximately 70%, dogs and cats were the most frequently presented animals on the analysed profiles in the two cities taken together. Gray et al. [17] emphasise that dogs are particularly popular on online dating apps because they are more social and require more care than cats, meaning that they underline the social skills of the user. Comparison of the cities indicates that more dogs are shown in Vienna than are shown in Tokyo and that more cats are shown in Tokyo than are shown in Vienna. This result is in line with our expectations and echoes other study results. In one such study, based on data gained from 22 Mio Tinder^®^ profiles in 16 countries, it was found that Austrian users presented dogs significantly more often they presented cats [33]. However, this data has to be interpretated with caution since it involved automised analysis using artificial intelligence software.

In general, the number of dogs and cats in modern societies’ households, as well as the intensification of relationships between these pets and their owners, is mirrored in the high prevalence of dogs and cats displayed on profiles showing animals. One explanation for this could be that our data were collected in urban areas, and hence the users had little direct contact with farm animals or wild animals. Perhaps in rural regions, the prevalence of animals beyond dogs and cats would rise in comparison to urban areas. Profiles that display particularly “unusual” animals such as wildlife or zoo animals, which might represent the more extraordinary life, play a rather vanishing role. With this in mind, it seems fair to say that the animals presented on profiles on the analysed app are mostly those with whom users frequently have direct and/or emotionally close contact. These offer insights into the everyday life of the user.

Turning to the relationship between socio-demographic factors and whether or not online dating users show animals on their profiles, our results indicate that gender, age and users’ cultural background (Tokyo as compared with Vienna) all have an impact on the probability of animals being shown. Thus, we found that female and older users are more likely than men and younger users to show animals on their profile photos. Although some of the animals (most obviously, wild creatures) would not have been owned, in general we did not gather information allowing us to determine whether the displayed animals (principally, the dogs and cats) were actually owned by the users. However, our findings are in line with other study results. For instance, a European study of pet owners has found that women and older adults are more likely than men and younger people to keep animals [21,22]. A possible explanation for this is that older adults often maintain lifestyles that make keeping animals easier. Younger people are more likely to want, or need, to have the flexibility to, for example, move between different cities. They may live in small apartments together with other people, or have no regular income to shoulder the costs of keeping a dog or cat. Sexual orientation did not show any significant differences in the analyses we conducted.

As regards possible cultural differences between users in Tokyo and Vienna, we found that significantly more users in Vienna than users in Tokyo had profiles with animals. Another significant difference was that only the Viennese profiles showed farm animals on the first profile photo. These differences can be plausibly explained by the urban structure of the cities. With around 10 million people, Tokyo has far more inhabitants than Vienna, which has around 2 million. It is also larger geographically (628 km^2^, as against Vienna’s 415 km^2^), which means that users in Tokyo tend to be further away from rural areas. Again, the population density in Tokyo is far higher (15,351/km^2^, as against Vienna’s 43,261/km^2^), suggesting that housing conditions there are more cramped and less pet-friendly. This last point may also help to explain why users in Tokyo are more likely than users in Vienna to display photos of cats, while dogs are more popular on Viennese profiles than they are on the profiles of users in Tokyo. Vienna—as we touched upon in the introduction—is the more dog-friendly of the two cities. However, the urban structure of Vienna and Japan may not be the only explanation for this difference, but rather general cultural differences between the two cities are decisive for the different prevalence of dogs and cats in profiles. For instance, it might be that dogs are generally more popular in Austrian culture or are ascribed a higher social status than in Japan. Nevertheless, where socio-demographic factors are concerned, we see that choices about the presentation of animals on the analysed app have close connections with everyday human–animal relationships. Online dating seems to be like a mirror of everyday human–animal relationships.

Self-presentation in online-dating is rarely spontaneous. In most cases it is a consciously planned activity [4,5]. It seems reasonable to infer that in most cases the inclusion of an animal photo is considered carefully the user. We might say that it is part of the user’s “impression construction” [5], by which we mean that users think about what impression they want to make and how they can achieve it. However, it is not only the fact that an animal is shown that seems to be relevant in this context, but also how users present animals. Our qualitative notes provide exploratory insights into this issue. In particular, they indicate an interweaving of “real life” and the use of the investigated dating app that manifests itself in various ways. Thus, the two typical presentations of animals (including the sub-categories) that we set out above in Section 3.5 correspond with the two key types of photo elaborated by Degen and Kleeberg-Niepage [4] in their qualitative, serial analysis of Tinder^®^ profile photos. According to the authors, so-called “informative type” photos offer insights into hobbies, activities and lifestyle. “Type of sociability and enjoyment” photos, by contrast, show the user interacting or in a social context [4]. In both cases, animals seem to be a great vehicle for conveying something of the user’s lifestyle and/or personality. With this mind, we might say that the displaying of animal photos serves to communicate aspects of the user—such as being empathic, kind, social, active, healthy, sporty, aesthetic or humorous—that play an important role in online dating for users, whether those users have long-term or short-term dating goals [34,35].

We believe that the present observational study provides useful preliminary insights into the ways different species of animal are displayed on the selected online dating app, and how these animals are presented on profile photos. However, we also recognise that the study has several limitations. We considered a sample size of a total of 2400 profiles including 300 profiles per research profile as sufficient in order to conduct analyses and provide first results on the outlined research questions. However, we suggest that future research may increase the number of profiles that can be investigated by research profiles. Further, the eight research profiles we set up meant that the study included only users of a specific app of a certain age group (20–40 years). Younger, as well as older, users as well users of other online dating apps were not investigated. Further, we decided to examine possible cultural differences by looking at just two cities—Vienna and Tokyo. We are aware that these two cities do not permit overall conclusions to be drawn about cultural differences more generally. In addition, the data were, of course, collected in urban regions. Hence, profiles in sub-urban as well as rural areas are yet to be investigated. As a consequence, we strongly recommend that future studies on this issue should, if possible, examine cities with different cultural backgrounds (e.g., in the US or Africa), consider not only urban, but also sub-urban and rural areas, and include profiles of younger (under 20 years) and older users (over 40 years) as well as users of other online dating apps. In addition, there may exist further aspects that influence the sampling of profiles that go beyond the defined search criteria used in this study. For instance, the number of “likes” a user receive can play a role. This aspect may result in a potential bias and sampling error of profiles investigated in this observational study. 

## 5. Conclusions

Animals do play a role in online-dating. Around 16% of the investigated profiles on the selected app displayed at least one animal. However, based on the high level of media interest in this topic, the percentage of animals in profile photos might have been expected to be higher. In both investigated cities (Tokyo and Vienna), dogs were the most frequently shown animal, followed by cats. Users in Vienna, women and older adults were more likely to present animals on their profiles than were users in Tokyo, men and younger users. Sexual orientation showed no significant differences in the analyses. We conclude that the animals most likely to be shown are those in a close and frequent contact with users (i.e., certain kinds of companion animal). Given that self-presentation in online-dating is usually not a spontaneous act but a consciously planned one, future studies could explore in depth the ways in which animals are represented and arranged on profile photos.

## Figures and Tables

**Table 1:** Characteristics of the eight researcher profiles.

Profile	City	Name	Gender	Sexual Orientation	Age (in Years)
1	Vienna	Andreas	male	heterosexual	30
2	Vienna	Eva	female	heterosexual	30
3	Vienna	Peter	male	homosexual	30
4	Vienna	Jenny	female	homosexual	30
5	Tokyo	Aiko	male	heterosexual	30
6	Tokyo	Akane	female	heterosexual	30
7	Tokyo	Benjiro	male	homosexual	30
8	Tokyo	Hina	female	homosexual	30

**Table 2:** Search criteria based on the eight researcher profiles.

	Criteria for Profiles 1 and 5	Criteria for Profiles 2 and 6	Criteria for Profiles 3 and 7	Criteria for Profiles 4 and 8
gender	female	male	male	female
sexual orientation	heterosexual	heterosexual	homosexual	homosexual
age (in years)	20–40	20–40	20–40	20–40
distance	50 km around Vienna or Tokyo	50 km around Vienna or Tokyo	50 km around Vienna or Tokyo	50 km around Vienna or Tokyo

**Table 3:** Set of questions in section B in relation to animals presented on profiles.

	Question	Answer Option
1	Is (are) there (an) animal(s) on any of the photos?	1 = yes 2 = no
2	Is there an animal (or animals) on the first profile photo?	1 = yes 2 = no
2.1	Classification of animal species shown on the first profile photo:	1 = Dog; 2 = Cat; 3 = Small animals (rodents/rabbits); 4 = Horse; 5 = Cagebird; 6 = Aquarium fishes; 7 = Terrarium animals; 8 = Farm animals; 9 = Wild animal; 10 = Exotic animals; 11 = Meme 12 = Other
3	In total, how many photos are photos with an animal or animals?	Answer option 1 to 10.
3.1	Classification of animal species shown on all profile photos:	1 = Dog; 2 = Cat; 3 = Small animals (rodents/rabbits); 4 = Horse; 5 = Cagebird; 6 = Aquarium fishes; 7 = Terrarium animals; 8 = Farm animals; 9 = Wild animal; 10 = Exotic animals; 11 = Meme 12 = Other

**Table 4:** Socio-demographic data and number of profile photos presented for all analysed profiles (*n* = 2400) and separately for Vienna (*n* = 1200) and Tokyo (*n* = 1200).

	All (*n* = 2342–2400)	Vienna (*n* = 200–1200)	Tokyo (*n* = 155–1200)	Tests
Gender				
Male	1200 (50.0)	600 (50.0)	600 (50.0)	
Female	1200 (50.0)	600 (50.0)	600 (50.0)	
Sexual orientation				
Heterosexual	1200 (50.0)	600 (50.0)	600 (50.0)	
Homosexual	1200 (50.0)	600 (50.0)	600 (50.0)	
Age in groups (in years)				U(565,676.5), *z* = −7.768, *p* < 0.001
20–25	748 (31.9)	262 (22.2)	486 (41.8)	
26–30	958 (40.9)	567 (48.1)	391 (33.6)	
31–35	477 (20.4)	264 (22.4)	213 (18.3)	
36–40	159 (6.8)	85 (7.2)	74 (6.4)	
Average age (in years)				U(423,937) *z* = −17.516, *p* < 0.001
Mean ± SD ^1^	27.7 ± 4.9	28.6 ± 4.5	27.0 ± 5.1	
Median age (in years)				
Median [IQR ^2^]	28 [24;31]	29 [16;31]	27 [22;30]	
Average number profile photos				U(4239,937), *z* = −17,516 *p* < 0.001
Mean ± SD	4.3 ± 2.38	5.1 ± 2.34	3.48 ± 2.12	
Median number profile photos				
Median [IQR]	4 [2;6]	5 [3;7]	3 [2;5]	

^1^ SD (standard deviation); ^2^ IQR (interquartile range); z-score Mann–Whitney-U Test.

**Table 5:** Socio-demographic data and number of profile photos presented for all profiles with animals (*n* = 373) and profiles without animals (*n* = 2027).

	Profiles with Animals (*n* = 363–373)	Profiles without Animals (*n* = 1979–2027)	Test
Gender			χ^2^(1) = 3.889, *p* = 0.049
Male	169 (45.3)	1031 (50.9)	
Female	204 (54.7)	996 (49.1)	
City			χ^2^(1) = 7.622, *p* = 0.006
Vienna	211 (56.6)	989 (48.8)	
Tokyo	162 (43.4)	1038 (51.2)	
Sexual orientation			χ^2^(1) = 0.156, *p* = 0.693
Heterosexual	190 (50.9)	1010 (49.8)	
Homosexual	183 (49.1)	1017 (50.2)	
Age in groups (in years)			U(328,248), *z* = −2.769, *p* = 0.006
20–25	88 (24.2)	660 (33.4)	
26–30	168 (46.3)	790 (39.9	
31–35	79 (21.8)	398 (20.1)	
36–40	28 (7.7)	131 (6.6)	
Average age (in years) (missing values = 58)			U(331,396), *z* = −2.354, *p* = 0.019
Mean ± SD ^1^	28.3 ± 4.75	27.7 ± 4.91	
Median age (in years)			
Median [IQR ^2^]	29 [26;31]	28 [24;31]	
Average number profile photos			U(250,389.5), *z* = −10.325, *p* < 0.001
Mean ± SD	5.43 ± 2.27	4.09 ± 2.34	
Median number profile photos			
Median [IQR]	5 [4;7]	4 [2;6]	

^1^ SD (standard deviation); ^2^ IQR (interquartile range); z-score Mann–Whitney-U Test.

## Data Availability

All relevant data are within the paper and its Supporting information files.

## Supplementary Materials

The following supporting information can be downloaded at: https://www.mdpi.com/article/10.3390/ani12030230/s1, Table S1: Binary regression analyses of socio-demographic aspects (including city, gender, sexual orientation, age) as well as number of profile photos on (i) displaying (an) animal(s) on photos in general, (ii) displaying (an) animal(s) on the first profile photo and (iii) displaying a cat or dog on profile photo.
