# Supplementary material for: Wanna See My Dog Pic? A Comparative Observational Study of the Presentation of Animals on Online Dating Profiles in Vienna and Tokyo
Source: Animals (Basel). 2022 Jan 19;12(3):230. doi: 10.3390/ani12030230 (PMC8833676; doi:10.3390/ani12030230)
Supplement: Supplementary file 1 [file animals-12-00230-s001.zip › animals-1507142-supplementary.pdf]

Supplementary File

Table S1. Binary regression analyses of socio-demographic aspects (including city, gender, sexual orientation, age) as well as number of profile photos on (i) displaying (an) animal(s) on photos in general, (ii) displaying (an) animal(s) on the first profile photo and (iii) displaying a cat or dog on profile photo.

**Model 1: Displaying (an) animal(s) on photos in general** ($\chi^2(5)$=102.495 p<0.001)					
	**B**	**Std. Error**	**Wald Ch-Square**	**df**	**Sig.**
(Intercept)	-3.197	0.4046	62.447	1	0.000
City (ref.cat.: Tokyo)	-0.118	0.1258	0.877	1	0.349
Gender (ref. cat.: female	-0.238	0.1201	3.942	1	0.047
Sexual orientation (ref. cat.: homosexual)	0.185	0.1202	2.364	1	0.124
Age	0.017	0.0128	1.700	1	0.192
Number of profile photos	0.236	0.253	86.798	1	0.000
**Model 2: Displaying (an) animal(s) on the first profile photo** ($\chi^2(5)$=30.147 p<0.001)					
	**B**	**Std. Error**	**Wald Ch-Square**	**df**	**Sig.**
(Intercept)	-2.711	0.9762	7.713	1	0.005
City (ref.cat.: Tokyo)	0.208	0.2932	0.505	1	0.477
Gender (ref. cat.: female	-0.244	0.2840	0.741	1	0.389
Sexual orientation (ref. cat.: homosexual)	0.054	0.2802	0.037	1	0.847
Age	0.094	0.0307	9.315	1	0.002
Number of profile photos	-0.282	0.0676	17.462	1	0.000
**Model 3: Displaying a cat or dog on profile photo** ($\chi^2(5)$=22.435 p<0.001)					
	**B**	**Std. Error**	**Wald Ch-Square**	**df**	**Sig.**
(Intercept)	-1.388	0.9673	2.058	1	0.151
City (ref.cat.: Tokyo)	1.117	0.2956	14.281	1	0.000
Gender (ref. cat.: female	0.504	0.2851	3.123	1	0.077
Sexual orientation (ref. cat.: homosexual)	0.497	0.2894	2.948	1	0.86
Age	0.033	0.0308	1.140	1	0.286
Number of profile photos	-0.008	0.0662	0.014	1	0.907
